# Effects of Glucagon-like Peptide-1 on the Reproductive Axis in Healthy Men

**DOI:** 10.1210/clinem/dgaa072

**Published:** 2020-02-13

**Authors:** Chioma Izzi-Engbeaya, Sophie Jones, Yoshibye Crustna, Pratibha C Machenahalli, Deborah Papadopoulou, Manish Modi, Christos Panayi, Jessica Starikova, Pei Chia Eng, Maria Phylactou, Edouard Mills, Lisa Yang, Risheka Ratnasabapathy, Mark Sykes, Isabella Plumptre, Ben Coumbe, Victoria C Wing, Ewa Pacuszka, Paul Bech, James Minnion, George Tharakan, Tricia Tan, Johannes Veldhuis, Ali Abbara, Alexander N Comninos, Waljit S Dhillo

**Affiliations:** 1 Section of Endocrinology and Investigative Medicine, Department of Medicine, Imperial College London, London, UK; 2 Department of Endocrinology, Imperial College Healthcare NHS Trust, London, UK; 3 Department of Acute Medicine, Imperial College Healthcare NHS Trust, London, UK; 4 Department of Internal Medicine, Mayo Clinic, Rochester, Minnesota

**Keywords:** glucagon-like peptide-1, luteinizing hormone, follicle stimulating hormone, testosterone, reproduction

## Abstract

**Context:**

Glucagon-like peptide-1 (GLP-1) potently reduces food intake and augments glucose-stimulated insulin secretion. Recent animal data suggest that GLP-1 may also influence reproduction. As GLP-1 receptor agonists are currently widely used in clinical practice to treat obesity/type 2 diabetes, it is necessary to determine the effects of GLP-1 on the reproductive system in humans.

**Objective:**

To investigate the effects of GLP-1 administration on the reproductive axis in humans.

**Design:**

Single-blind, randomized, placebo-controlled crossover study.

**Setting:**

Clinical Research Facility, Imperial College Healthcare NHS Trust.

**Participants:**

Eighteen healthy men (mean age 24.7 ± 0.1years, mean BMI 22.1 ± 0.4kg/m^2^).

**Intervention:**

Eight-hour intravenous infusion of 0.8 pmol/kg/min GLP-1 or rate-matched vehicle infusion.

**Main Outcome Measures:**

Number of luteinizing hormone (LH) pulses, LH, follicle-stimulating hormone (FSH), and testosterone levels.

**Results:**

The number of LH pulses (number of LH pulses/500 min: vehicle 4.2 ± 0.4, GLP-1 4.5 ± 0.3, *P* = 0.46), LH area under the curve (AUC) (vehicle 1518 ± 88min.IU/L, GLP-1 1524 ± 101min.IU/L, *P* = 0.95), follicle-stimulating hormone AUC (vehicle 1210 ± 112 min IU/L, GLP-1 1216 ± 112 min IU/L, *P* = 0.86), and testosterone AUC (vehicle 10893 ± 615 min nmol/L, GLP-1 11088 ± 792 min nmol/L, *P* = 0.77) did not significantly differ during vehicle and GLP-1 administration. Glucagon-like peptide-1 significantly reduced food intake (vehicle 15.7 ± 1.3 kcal/kg, GLP-1 13.4 ± 1.3 kcal/kg, *P* = 0.01).

**Conclusions:**

In contrast to the animal literature, our data demonstrate that acute GLP-1 administration does not affect reproductive hormone secretion in healthy men.

Reproduction and metabolism are interconnected, but the mediators of the interaction between these physiological processes are poorly understood (1). Up to 40% of men with obesity and/or type 2 diabetes have co-existing hypogonadism, which is associated with an adverse metabolic phenotype (1). Testosterone treatment improves hypogonadism, but there are ongoing safety concerns limiting its use (2). As the prevalence of obesity/diabetes rises, alternative strategies for managing co-existing obesity/diabetes and hypogonadism are required.

Glucagon-like peptide-1 (GLP-1), produced by intestinal L-cells postprandially (3), potently reduces food intake, induces weight-loss, and augments insulin secretion (4). Glucagon-like peptide-1 receptor agonists (GLP-1RAs) are currently used to treat obesity/type 2 diabetes. Glucagon-like peptide-1 receptor agonism may also have regulatory effects on the reproductive system (5–8). Glucagon-like peptide-1 administration increases hypothalamic kisspeptin (a key regulator of hypothalamic gonadotropin-releasing hormone [GnRH] secretion) expression (5) and stimulates GnRH secretion from rodent hypothalamic explants (6). Furthermore, liraglutide, a GLP-1RA, increases kisspeptin neuronal firing (7). In female rats, GLP-1 increases the preovulatory luteinizing hormone (LH) surge (8), but exendin-4 (another GLP-1RA) reduces LH levels (8).

Acute infusion of GLP-1 to healthy men during a euglycemic clamp (maintaining circulating glucose levels around 5 mmol/L) does not alter mean reproductive hormone levels; but it reduces the number of testosterone pulses with a trend towards longer testosterone pulse duration (9). However, chronic administration of liraglutide to obese, hypogonadal men with type 2 diabetes, increases testosterone to a greater extent than testosterone treatment and metformin alone (10). In obese hypogonadal men, 16 weeks of liraglutide treatment increases reproductive hormone levels (11). It is unclear if GLP-1 receptor agonism has a beneficial effect on the reproductive axis in the absence of weight loss, as these studies reported significant weight loss in the groups that received liraglutide (10, 11). Additionally, in the study reporting no effect of acute GLP-1 infusion on reproductive hormone levels (9), a subanorectic dose of GLP-1 was used (12). Consequently, this dose may have been too low to affect reproductive hormone levels.

Therefore, we performed a single-blind, randomized, placebo-controlled crossover study of administration of a biologically active dose of GLP-1 to healthy men to test the hypothesis that GLP-1 has direct effects on the reproductive axis.

## Materials and Methods

### Study participants

This study was performed in accordance with the Declaration of Helsinki and received approval from the West London Research Ethics Committee (16/LO/0391). Recruitment via advertisements took place between November 2017 and August 2018. Eighteen healthy men (age 24.7 ± 1years, BMI 22.1 ± 0.4 kg/m^2^, baseline testosterone 21.9 ± 1.5 nmol/L and calculated free testosterone (13) 0.52 ± 0.03 nmol/L) were enrolled in the study after confirmation of eligibility (ie, absence of active medical or psychiatric conditions and no use of prescription drugs, recreational drugs, and nicotine-containing products within the preceding 3 months) and with the provision of written informed consent.

### Study visits

All participants attended 2 study visits, 1 for GLP-1 administration and 1 for vehicle administration. Infusion order was randomized and participants were blinded as to the identity of the infusions. GLP-1_7–36_ was infused at a rate of 0.8 pmol/kg/min, a dose established to reduce food intake in humans (4). Rate-matched vehicle infusions were comprised of Gelofusine (Braun, Sheffield, UK) only.

**Figure 1.**
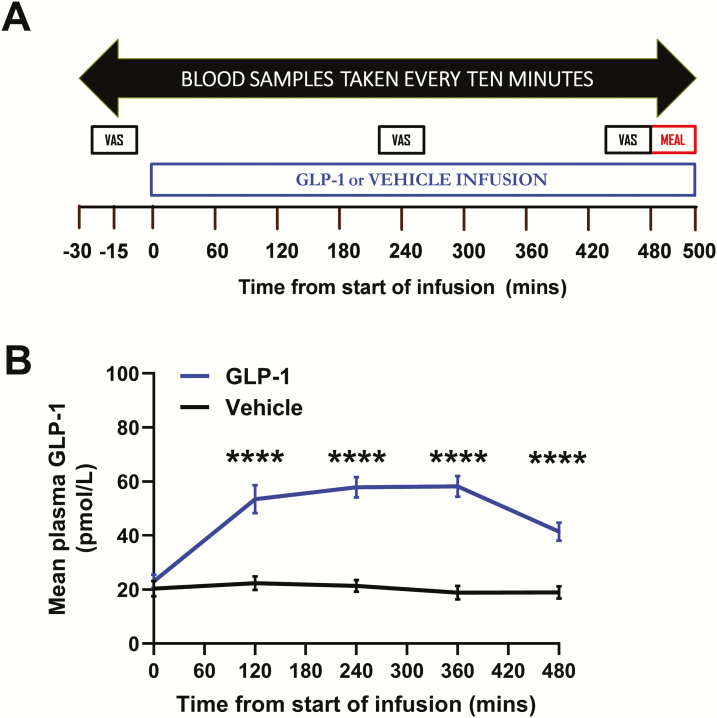
Study protocol and GLP-1 levels. **A:** After an overnight fast and a standardized breakfast, 18 healthy men attended 2 study visits, one with 0.8 pmol/kg/min glucagon-like peptide-1 (GLP-1) infusion and one with (rate-matched) vehicle infusion for 500 minutes. The order of the infusions was randomly determined. Blood samples were taken at 10 minute intervals throughout each study visit (apart from during the *ad libitum* meal). Visual analogue scales (VAS) were completed by participants to assess subjective nausea preinfusion (at T = -15 minutes), midinfusion (at T = 240 minutes), and premeal (at T = 470mins). An *ad libitum* meal was given to the participants at T = 480 minutes. **B:** Plasma GLP-1 levels were higher during GLP-1 infusion compared to vehicle infusion. Two-way repeated measures analysis of variance (RM-ANOVA) detected a significant interaction of treatment (ie, vehicle vs. GLP-1) and time (*P* < 0.0001). Asterisks indicate significant differences at specific timepoints (****P < 0.0001).

On each study visit, following an overnight fast, each participant ate a standardised 200 kcal breakfast at 6:00 am and arrived at the clinical research facility at 8.15 am. Two intravenous cannulae were inserted (1 in each arm; 1 cannula was used to administer the infusion and the other cannula was used to obtain blood samples). After baseline sampling, GLP-1/vehicle infusion was started at T = 0 minutes and continued until T = 500 minutes. Visual analogue scales (VAS, 0–10 cm) were used to measure participants’ self-reported nausea at T = -15 minutes, T = 240 minutes, and T = 470 minutes. Participants were given an *ad libitum* meal at T = 480 minutes. Blood samples were taken every 10 minutes (Fig. 1A).

### Biochemical analyses

Plasma glucose, serum insulin, LH, follicle-stimulating hormone (FSH), and testosterone were measured (in single sample aliquots) by NorthWest London Pathology on the automated Abbott Architect® platform. Chemiluminescent immunoassays were used to measure serum insulin (intra-assay and interassay coefficient of variation [CV]: ≤7%), serum LH (intra-assay and interassay CV: ≤5%), serum FSH (intra-assay and interassay CV: ≤10%), and serum testosterone (intra-assay and interassay CV: ≤8%). Plasma glucose was measured using a colorimetric hexokinase assay (intra-assay and interassay CV: ≤2%). Total plasma GLP-1 was measured (in duplicate) using an in-house radioimmunoassay (intra-assay and interassay CV: ≤10%) utilizing an antibody that detects GLP-1_7-36amide_ and GLP-1_9-36amide_ but not glycine-extended forms of GLP-1 (3).

### Statistical methods

Based on existing literature (14), a sample size of 18 men provides 90% power to detect a difference in LH (between vehicle and GLP-1 infusion) of 2 IU/L (SD 2.3 IU/L) at a significance level of 0.05. Data from all 18 participants were included in the analyses. Luteinizing hormone pulsatility was determined using a validated blinded deconvolution analysis (15). The differences in hormone levels and nausea during vehicle infusion compared with GLP-1 infusion were compared using a 2-way repeated measures analysis of variance (RM-ANOVA) with Bonferroni’s post hoc multiple correction tests for individual timepoint comparisons.

Luteinizing hormone, FSH, and testosterone areas under the curve (AUCs) were calculated using the trapezoidal rule (16). Hormone AUCs and food intake data were compared using paired t-tests. Statistical analyses were performed using STATA 14.1 (STATACorp, College Station, TX, USA) and Prism 8.0.2 (GraphPad, San Diego, CA, USA) software. *P*-values <0.05 were considered statistically significant. Data are presented as mean ± SEM.

## Results

### Effects of GLP-1 on LH, FSH, and testosterone

**Figure 2.**
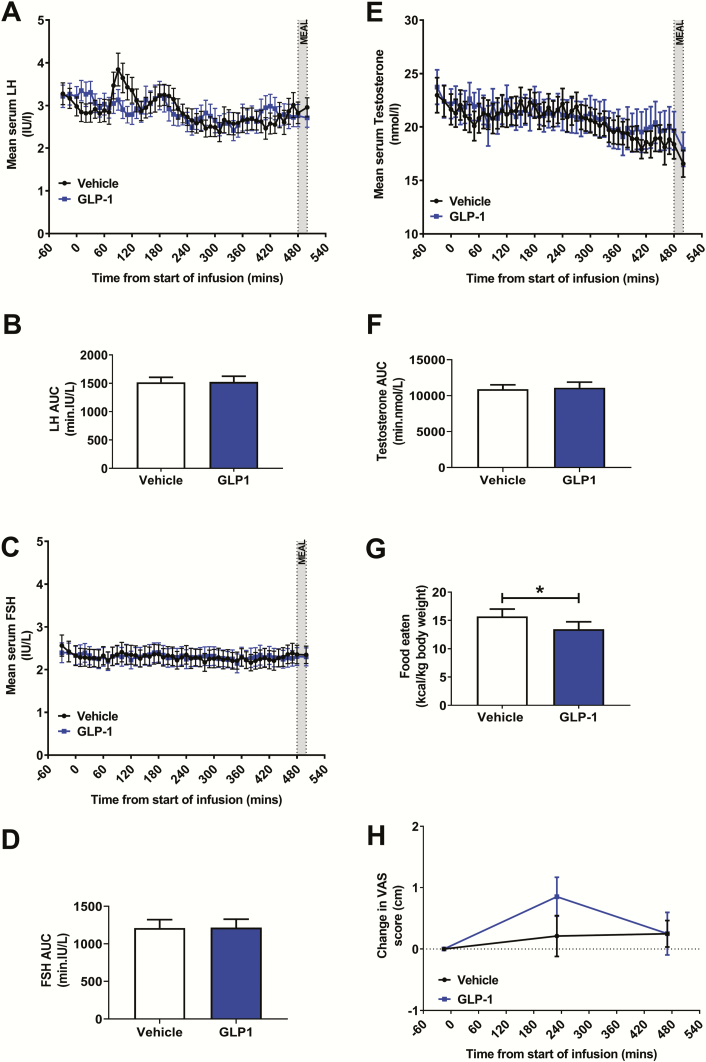
Effects of GLP-1 infusion on reproductive hormone levels, food intake, and nausea. **A:** Mean serum luteinizing hormone (LH) levels were similar during GLP-1 and vehicle infusions. Two-way RM-ANOVA did not detect a significant interaction of treatment (vehicle vs. GLP-1) and time (*P* = 0.16). MEAL = *ad libitum* meal. **B:** There was no significant difference between LH area under the curve (AUC) during GLP-1 infusion compared with vehicle infusion (*P* = 0.95 using paired t-test). **C:** Mean serum follicle stimulating hormone (FSH) levels were similar during GLP-1 and vehicle infusions. Two-way RM-ANOVA did not detect a significant interaction of treatment (vehicle vs. GLP-1) and time (*P* = 0.29). MEAL = *ad libitum* meal. **D:** There was no significant difference between FSH area under the curve (AUC) during GLP-1 infusion compared with vehicle infusion (*P* = 0.86 using paired t-test). **E:** Mean serum testosterone levels were similar during GLP-1 and vehicle infusions. Two-way RM-ANOVA did not detect a significant interaction of treatment (vehicle vs. GLP-1) and time (*P* = 0.71). MEAL = *ad libitum* meal. **F:** There was no significant difference between testosterone area under the curve (AUC) during GLP-1 infusion compared with vehicle infusion (*P* = 0.77 using paired t-test). **G:** Food intake was lower during GLP-1 infusion compared to rate-matched vehicle infusion (body weight-adjusted food intake at T = 480 mins: vehicle 15.7 ± 1.3 kcal/kg vs. GLP-1 13.4 ± 1.3 kcal/kg, *P = 0.01 using paired t-test). **H:** Using visual analogue scales (VAS, 0–10 cm), there was no significant difference in participants’ self-reported (change from baseline) nausea during GLP-1 infusion compared with vehicle infusion. Two-way RM-ANOVA did not detect a significant interaction of treatment (vehicle vs. GLP-1) and time (*P* = 0.26).

Glucagon-like peptide-1 administration resulted in elevated GLP-1 levels (Fig. 1B). However, there were no significant differences between serum LH levels and LH area under the curve (AUC) during vehicle and GLP-1 administration (Fig. 2A and 2B). Furthermore, GLP-1 administration did not alter the number of LH pulses (number of LH pulses/500 min: vehicle 4.2 ± 0.4, GLP-1 4.5 ± 0.3, *P* = 0.46) nor the mean LH pulse mass (vehicle 5.7 ± 0.7 IU/L, GLP-1 4.6 ± 0.6 IU/L, *P* = 0.26).

There were no significant differences between serum FSH levels and FSH AUC during vehicle and GLP-1 administration (Fig. 2C and 2D). Intravenous GLP-1 administration did not alter serum testosterone levels, did not affect the diurnal variation in testosterone levels, and did not affect testosterone AUC (Fig. 2E and 2F). Additionally, GLP-1 administration did not affect testosterone pulsatility (number of testosterone pulses/500 mins: vehicle 4.3 ± 0.6 vs. GLP-1 4.6 ± 0.4, *P* = 0.76).

### Effects of GLP-1 on glucose, food intake, and nausea

Compared to vehicle administration, plasma glucose levels were lower during GLP-1 administration (vehicle 4.99 ± 0.05 mmol/L vs. GLP-1 4.66 ± 0.06 mmol/L, *P* < 0.0001). Participants were given an *ad libitum* meal at T = 480 minutes and intravenous administration of GLP-1 resulted in 15% reduction in food intake (Fig. 2G). Therefore the dose of GLP-1 administered was biologically active. Glucagon-like peptide-1 receptor agonists cause dose-dependent nausea (17). Nausea (similar to other types of stress (18)) may have an inhibitory effect on reproductive hormone release. Therefore, we assessed the participants’ self-reported nausea using a 0 to10 cm visual analogue scale (VAS) preinfusion, midinfusion, and just prior to the *ad libitum* meal (Fig. 1A). Glucagon-like peptide-1 infusion resulted in similar nausea VAS ratings to vehicle (Fig. 2H).

## Discussion

This is the first study investigating the effect of high-dose GLP-1 infusion on reproductive hormone secretion in humans. Our data demonstrate that intravenous infusion of GLP-1, administered at a rate of 0.8 pmol/kg/min for 500 minutes, reduces food intake but does not alter serum levels of reproductive hormones in young healthy men. Fertility is dependent on absolute reproductive hormone levels as well as LH pulsatility (19). Therefore, we also assessed the effect of GLP-1 on LH and testosterone pulsatility and demonstrate that GLP-1 administration does not affect LH and testosterone pulsatility. Our results are in agreement with some published human data but not with the animal literature.

In rodents, both stimulatory and inhibitory effects of GLP-1 receptor agonism on reproductive hormones have been reported (5, 7, 8). This might be due to LH-dependent and/or LH-independent mechanisms. For instance, in rodents, testosterone production is suppressed by central administration of norepineprine (20); intravenous administration of a GLP-1RA induces cFos immunoreactivity in catecholamine neurons and increases expression of tyrosine hydroxylase (the enzyme that catalyzes the rate-limiting step of the catecholamine synthesis pathway) (21). However, chronic central administration of a GLP-1RA to rodents reduces urinary norepinephrine secretion (ie, a surrogate marker of norepinephrine production) (22). Therefore, there may be divergent effects of acute versus chronic GLP-1 receptor agonism on catecholamines and testosterone. In the current study, acute administration of GLP-1 to healthy men did not affect testosterone secretion and pulsatility; other groups have reported that chronic administration of GLP-1 receptor agonists increases testosterone levels in obese hypogonadal men (10, 11). Thus, unlike in rodents, acute and chronic GLP-1 receptor agonism does not appear to have adverse effects on testosterone secretion in humans. The reason for this species difference requires further mechanistic study.

In humans, a 6-hour continuous intravenous infusion of 0.4 pmol/kg/min GLP-1 to 9 healthy men during a euglycemic clamp did not affect LH pulsatility or mean serum LH, FSH, and testosterone levels, but GLP-1 administration reduced the number of testosterone pulses with a trend towards increased testosterone pulse duration (9). Volunteers remained euglycemic in both the clamp study (mean plasma glucose was 5 mmol/L during GLP-1 administration and 5.2 mmol/L during saline administration) (9) and in our study (mean plasma glucose was 4.66 mmol/L during GLP-1 administration and 4.99 mmol/L during vehicle administration). As testosterone pulses are not affected by glucose concentrations between 4 and 5.5mmol/L (23, 24), the use of euglycemic clamp methodology would not be expected to influence testosterone pulsatility. Additionally, the differing glucose levels between the two studies do not account for the difference in the effect of GLP-1 administration on testosterone pulsatility reported (ie, reduced number of pulses vs no effect on pulse number respectively). Administration of 0.4 pmol/kg/min GLP-1 does not reduce food intake (12); therefore, the lack of a significant effect on reproductive hormone levels reported may have been due to the low dose of GLP-1 used (9). However, we used a higher dose of GLP-1 (ie, 0.8 pmol/kg/min) that reduced food intake (without causing nausea) but did not alter LH pulsatility, testosterone pulsatility, and circulating reproductive hormone levels.

Increases in LH and testosterone levels in hypogonadal obese men with/without type 2 diabetes have been reported following long-term administration of GLP-1RAs (10, 11). In these studies, the men who received GLP-1RA lost more weight than people in the comparator groups. Weight loss is associated with improvement in reproductive hormone levels in obese men with hypogonadism (1). Consequently, the improvement in reproductive hormones reported in these studies may be due to weight loss produced by the GLP-1RAs and not due to a direct effect of GLP-1 receptor agonism.

Measurement of serum testosterone in this study was performed using a chemiluminescent immunoassay. While this is a well-established assay for the measurement of serum testosterone, using mass spectrometry would have provided greater accuracy. Additionally, in this study the participants consisted solely of healthy eugonadal men who received an acute GLP-1 infusion. Thus, the main limitation of this study is the lack of an obese control group and/or a lack of data on chronic use in healthy men (in contrast to the positive data with chronic liraglutide in obese men). Therefore, we cannot conclude with certainty that the beneficial reproductive effects with chronic GLP1-RA in obese hypogonadal men are due to concomitant weight loss or not. As the prevalence of obesity and type 2 diabetes is increasing in both men and women, and both men and women are receiving GLP-1RAs, further studies are required to determine the direct effects (if any) of GLP-1 and GLP-1RAs on reproductive hormone secretion in women and hypogonadal patients with obesity/diabetes.

## Conclusions

Our data demonstrate that in healthy eugonadal men, administration of a biologically active dose of GLP-1 has no effect on LH pulsatility and does not alter serum levels of LH, FSH, or testosterone. This important data contributes to our understanding of the interaction between metabolic and reproductive systems in humans.

## Additional Information:

***Disclosure Summary:*** The authors have nothing to disclose.
